# Myometrial progesterone hyper-responsiveness associated with increased risk of human uterine fibroids

**DOI:** 10.1186/s12905-019-0795-1

**Published:** 2019-07-09

**Authors:** Mona Omar, Archana Laknaur, Ayman Al-Hendy, Qiwei Yang

**Affiliations:** 1 Division of Translation Research, Department of Obstetrics and Gynecology, Augusta University, Medical College of Georgia, Augusta, GA USA; 2 Department of Obstetrics and Gynecology, Tanta University Faculty of Medicine, 3 El-Bahr Street, Tanta, Egypt; 3 Department of Obstetrics and Gynecology, University of Illinois at Chicago, 909 S. Wood Street, (M/C 808), Chicago, IL 60612 USA; 4 Department of Obstetrics and Gynecology, University of Illinois @ Chicago (UIC), 820 South Wood Street, Chicago, IL 60612 USA; 5 Georgia Cancer Center, Augusta University, 1410 Laney Walker Blvd, Augusta, GA 30912 USA

**Keywords:** Uterine fibroids, Progesterone, Progesterone-regulated genes, Progesterone receptor

## Abstract

**Background:**

Uterine Fibroids (UFs) growth is ovarian steroid-dependent. Previous studies have shown that estrogen and progesterone play an important role in UF development. However, the mechanism underlying progesterone induced UF pathogenesis is largely unknown. In this study, we determined the expression of progesterone receptor and compared the expression level of progesterone-regulated genes (PRGs) in human myometrial cells from normal uteri (MyoN) versus uteri with UFs (MyoF) in response to progesterone.

**Methods:**

Primary human myometrial cells were isolated from premenopausal patients with structurally normal uteri (PrMyoN). Primary human myometrial cells were also isolated from uterus with UFs (PrMyoF). Isolated tissues were excised at least 2 cm from the closest UFs lesion(s). Progesterone receptor (PR) expression was assessed using Western blot (WB). Expression levels of 15 PRGs were measured by qRT-PCR in PrMyoN and PrMyoF cells in the presence or absence of progesterone.

**Results:**

WB analysis revealed higher expression levels of PR in PrMyoF cells as compared to PrMyoN cells. Furthermore, we compared the expression patterns of 15 UF-related PRGs in PrMyoN and PrMyoF primary cells in response to progesterone hormone treatment. Our studies demonstrated that five PRGs including *Bcl2*, *FOXO1A, SCGB2A2, CYP26a1 and MMP11* exhibited significant progesterone-hyper-responsiveness in human PrMyoF cells as compared to PrMyoN cells (*P* < 0.05). Another seven PRGs, including *CIDEC, CANP6, ADHL5*, *ALDHA1, MT1E, KIK6, HHI* showed gain in repression in response to progesterone treatment (*P* > 0.05). Importantly, these genes play crucial roles in cell proliferation, apoptosis, cell cycle, tissue remodeling and tumorigenesis in the development of UFs.

**Conclusion:**

These data support the idea that progesterone acts as contributing mechanism in the origin of UFs. Identification and analysis of these PRGs will help to further understand the role of progesterone in UF development.

## Background

Uterine fibroids (UFs) are smooth muscle cell tumors originating from the myometrium. Tumors occur in 70–80% of women overall and are clinically manifested in 25–50% by 50 years of age [1].

UFs are ovarian steroid hormones dependent [2]. While estrogen has been considered the major mitogenic factor in the uterus, progesterone (P4) also play a key role in UF growth and development [3]. There are conflicting results about the role of progesterone in UF development either it is stimulatory or inhibitory [4]. Several studies had proved the essential role of the progesterone in the pathogenesis of UFs. A xenograft animal model demonstrated that estradiol upregulated the PR levels, and volume maintenance and growth of UFs were P4 dependent (Hiroshi Ishikawa et al. Endocrinology 2010 and For human studies,pregnancy stimulateed UF development / growth were suppressed with antiprogestin therapy [5]. Several other studies demonstrated that GnRH agonists were capable of reducing UF size, and progestin add-back therapy prevented this reduction [6–8]. In addition, LNG (levo –norgestrine) (treatment in vitro decreased UF cell viability and induced apoptosis. Similarly, a number of antiprogestin drugs and SPRM (selective progesterone receptor modulator) have been developed and tested in clinical trials for the treatment of UFs, including Mifepristone, Asoprisnil and Ulipristal acetate. These studies provide strong evidence for the mitogenic effect of progesterone on UF pathogenesis [9–11].

The progesterone responses are mediated by two pathways, the rapid non-genomic signaling and slower genomic one. The genomic pathway can be mediated by binding of progesterone to PR result in binding to DNA and regulate the expression of target genes. There are two types of PR, PR-A and PR-B [12–14]. PR-A and -B actions are divergent from each other. PR-B differs from PR-A in that it contains an additional 164 amino acids at the amino-terminus [15, 16]. Another mode of action are non-genomic pathway, in which the progesterone activate a variety of rapid signaling events in the cells [17].

Eker rat model carrying a germ-line defect in the tuberous sclerosis complex-2 (*Tsc-2*) tumor suppressor has been used to determine the interaction between genetic susceptibility and early-life environmental exposure, which contributes to the pathogenesis of UFs [18, 19]. Developmental exposure to xenoestrogens such as diethylstilbestrol (DES) increased the tumor-suppressor-gene penetrance, tumor multiplicity and size in predisposed animals, and DES exposure caused reprogramming of estrogen-response genes expressed in UFs and resulted in alteration of these genes in UFs tumor genesis. Recently, we demonstrated that developmental exposure to DES expands the myometrial stem cells (MMSCs), which linked to the increases risk of UF development. In human studies, the correlation between number of MMSCs with risk of UFs was identified in women. Myometrium from Caucasian (CC) women with UFs exhibited increased numbers of MMSCs as compared to CC women without UFs, and myometrium from African-American (AA) women had the highest number of MMSCs: AA-with UFs > CC with UFs > AA-without UFs > CC-without UFs [20]. In addition, MMSC population expanded in African American women, is correlated with parity and UF number, and fluctuates with cyclic menstrual cycle hormone changes and age [21, 22]. These studies suggest that MyoF was primed and exhibited a distinct profiling at molecular and cellular levels as compared to MyoN, which become at risk for later tumorigenic transformation. However, how the P4 triggers the transformation is unknown.

The object of this study is to identify progesterone responsive genes in cells from human MyoF verse MyoN tissues that will help to understand the role of progesterone in UF development.

## Methods

### Patients and myometrium specimens

The study was approved by Augusta University’s Institutional Review Board. Myometrium were obtained from Caucasian women who underwent abdominal hysterectomy for UFscause or any other causes. The ages of the patients are from 33 to 48 years old and none had received hormonal therapy for at least three cycles before surgery. The case interquartile range is 6. Informed consent was obtained from each patient before surgery for the use of extirpated uterine tissues for culture experiments.

### Myometrial cell isolation and cell culture

Primary human myometrial cells were generated from the adjacent myometrial tissue of human uterus with UF after hysterectomy at least 2 cm away from the closest UFs lesion (PrMyoF). Also we isolated the primary human myometrial cells from uterus without UFs (PrMyoN). Isolation of the primary cell population from tissues was performed as described previously [23]. Briefly, a portion (0.5 cm3) of fresh myometrial tissue was washed in culture medium to remove blood and then chopped into small pieces under sterile conditions, transferred into a 15-ml screw cap tube, and suspended in Hanks Balanced Salt Solution containing 13 antibiotic-antimycotic (Thermo Fisher Scientific) and 300 U/ml collagenase type 4 (Worthington Biochemical Corp.). Suspended tissue pieces were incubated at 37 °C for at least 12 h to obtain individual cells and/or clumps of cells. The cell suspension was passed through a 100-μm pore-sized sterile nylon filter and the suspension of individual cells was plated out and incubated at 37 °C, allowing the cells to attach to the 100-mm sterile tissue culture-treated plate containing smooth muscle cell basal medium (SmBM; catalog no. CC- 3181; Lonza) containing 5% fetal bovine serum (FBS) and supplemented with SmBM singlequots (catalog no. CC-4149). This SmBM singlequot contains hEGF, insulin, hFGF-B, and gentamicin / amphotericin-B. These cell culture experiments were performed successfully with ten uterine tissue specimens collected from different patients, of which five were from the normal uterus and the other five were from uterus with UFs.

### Protein extraction and Western blot analysis

Pellets were lysed in lysis buffer with protease and phosphatase inhibitor cocktail (Thermo Fisher scientific, Waltham, MA, USA), and protein was quantified using the Bradford method (Bio-Rad protein Assay kit, Hercules, CA, USA). Western blot was performed as described previously [24] Blots were done for two different isoforms of the progesterone receptors PR-A, PR-B. Both are polyclonal antibody used in dilution 1: 500 (Santa Cruz sc-7208, sc-538).

### Cell treatment

Primary myometrial cells were cultured in 60-mm dishes at 30–40% confluence at an approximate density of 5 × 10^5^ cells/dish at 37^0^ C in a humidified atmosphere of 5% CO_2_ in the regular SmBM media. When the cells were reached at approximately 80% confluence, the cells were grown in serum-free medium for 24 h. Then the cells were treated with P4 (1.0 ng/mL) for 72 h.

### Quantitative real-time PCR

RNA was isolated according to the protocol using RNeasy Mini Kit. Following RNA extraction, cDNA was made by Reverse-transcribing 1 μg of RNA using the (RNA to cDNA Eco Dry Premix (double Primed)). Aliquots of cDNA were made for each sample and stored at –20 °C until analyzed.

SYBR Green real-time PCR was performed as described previously [25]. Briefly, RNA expression of genes was detected using Sso Advanced Universal SYBR Green Supermix on a Bio-Rad CFX96 real-time PCR system. Data were analyzed using Bio-Rad CFX manager software. Each biological sample was run in triplicate for each individual experiment. All assays were carried out in 96-well format. Real-time fluorescent detection of PCR products was performed with the CF96X Real-Time PCR System (Bio-Rad) using the following thermocycling conditions: 1 cycle of 95 °C for 10 min; 40 cycles of 95 °C for 30 s, and 60 °C for 1 min. The primer sequences for qPCR were shown in Table 1 [26].

**Table 1:** List of primer sequences used for qPCR [26]

Gene	Name	Function	Forward primer (5–3)	Reverse primer (5--3)
FOXO1A	Forkhead box O1A	Transcription factor in induction of apoptosis	AAGAGCGTGCCCTACTTCAA	CTGTTGTTGTCCATGGATGC
Bcl2	B-cell CLL/lymphoma 2	Block apoptotic	AGTTATCGGCTTCAGTGGTCT	CTGCCCGCTTCCTAGCTTG
SCGB2A2	Secretoglobin, family 2A, member 2	A uteroglobin-related genes	ACCATGAAGTTGCTGATGGTC	GGCATTTGTAGTGGCATTGTC
		Control cell cycle and DNA replication.		
CIDE	cell death-inducing DFFA-like effector c	Induce apoptosis	AAGTCCCTTAGCCTTCTCTACC	CCTTCCTCACGCTTCGATCC
CAPN6	Calpain 6	It is calcium-activated cysteine proteinases	GGAAGCGTCCACAGGACATTT	TCATTGCCTTGTTCCCCAATC
CYP26a1	cytochrome P450, family 26, subfamily a, polypeptide1	RA catabolizing enzyme	AGAGCAATCAAGACAACAAGTTAG	ATCGCAGGGTCTCCTTAAT
ALDH1a1	Aldehyde dehydrogenase family 1, subfamily A1	RA synthesis enzymes	GCACGCCAGACTTACCTGTC	CCTCCTCAGTTGCAGGATTAAAG
ADH5	Aldehyde dehydrogenase family5	RA synthesis enzymes	ATGGCGAACGAGGTTATCAAG	CATGTCCCAAGATCACTGGAAAA
MT1E	Metallothionein 1E	Metallothioneins (MTs) family that bind to heavy metal ions and minimize reactive oxygen species.	GCAAGTGCAAAAAGTGCAAAT	CACTTCTCTGACGCCCCTTT
MT2A	Metallothionein 2A		TCGGACAAGTGCAGCTGCTG	CCCTCCCAGTTCAATCCCTC
MTG2	Metallothionein G2		TGGGACACAAACCTCAAATG	TGATGAGCCTATGCAGACAC
KlK6		Regulation of the inflammatory process	CCAAACTCTCTGAACTCATCCAG	GTGTCAGGGAAATCACCATCTG
HHI ()	Indian hedgehog	down regulation of cellular division	AACTCGCTGGCTATCTCGGT	GCCCTCATAATGCAGGGACT
Calcitonin		Reduce production of pro-inflammatory cytokines, protective factor in ischemia	CCTATCCAACAATAGAGCCCAAG	TGCATTCGGTCATAGCATTTGTA
MMP11	Matrix metalloproteinases	Regulate cell-cell interactions and release the growth factors	AGACACCAATGAGATTGCAC	GCACCTTGGAAGAACCAAATG

### Statistical analysis

All values are expressed as means ± SE. Comparisons between two groups were done using the unpaired Student t-test. Differences between groups were examined by ANOV. Values of *P*< 0.05 were considered statistically significant.

## Results

### Subject characteristics

All the samples were taken from the Caucasian women with age ranges 33–48 years old (median 41.5). Subject characteristics is shown in Table 2 (*n* = 10).

**Table 2:** Subject characteristics (*n* = 10)

Patient ID	Type of primary cell	Age	Ethnicity	Diagnosis
Subject 1	Pr MyoN	39	Caucasian	Prolapsed uterus
Subject 2	Pr MyoN	40	Caucasian	Prolapsed uterus
Subject 3	Pr MyoN	38	Caucasian	Prolapsed uterus
Subject 4	Pr MyoN	43	Caucasian	Prolapsed uterus
Subject 5	Pr MyoN	39	Caucasian	Prolapsed uterus
Subject 6	Pr MyoF	46	Caucasian	uterus with UFs
Subject 7	Pr MyoF	45	Caucasian	uterus with UFs
Subject 8	Pr MyoF	33	Caucasian	uterus with UFs
Subject 9	Pr MyoF	48	Caucasian	uterus with UFs
Subject 10	Pr MyoF	45	Caucasian	uterus with UFs

### Altered PR expression in MyoN and MyoF

**Fig. 1:**
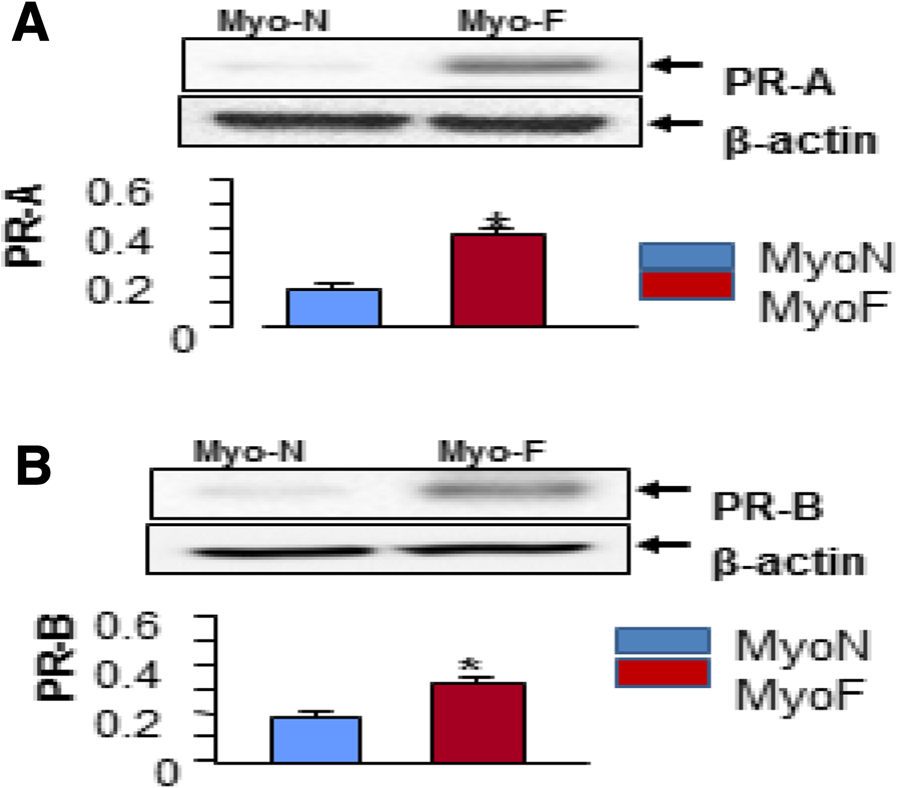
Western blot analysis of progesterone receptor A and B in MyoF and MyoN cells. Western blot analysis of proteins extracted from normal myometrium cells in normal uterus (MyoN) (*n* = 5) or myometrium of uterus with UFs (MyoF, *n* = 5) was performed. Total lysates from myometrial tissues were extracted and subjected to Western blot analysis using antibodies against Progesterone receptor A (**a**) and Progesterone receptor B respectively (**b**). β-actin was used as an endogenous control. A statistically significant increase in expression of PR-A was observed in PrMyoF cells as compared to PrMyoN cells (**a**, *P* < 0.05). **b** showed a statistically significant increase in expression of PR-B in PrMyoF compared to PrMyoN cells

To determine if MyoN and MyoF exhibited differential gene expression pattern in response to P4 treatment, we first examined the expression of both PR-A, PR-B in MyoN and MyoF primary cells. Western blot (WB) analysis showed that the expression levels of PR-A were significantly higher in PrMyoF as compared to PrMyoN) (Fig. 1a). The similar result was achieved for the PR-B (Fig. 1b). We confirmed the result in other patients in our experiment (*P* < 0.05).

### Genes show gain of induction in response to progesterone (P4)

Previous studies have identified various progesterone target genes in endometriosis or during menstrual cycle [27–29]. In this study, we selected 15 UF-related genes and determined their differential expression between PrMyoF and PrMyoN cells in the presence or absence of the P4 (1.0 ng/ml) by qRT-PCR.

**Fig. 2:**
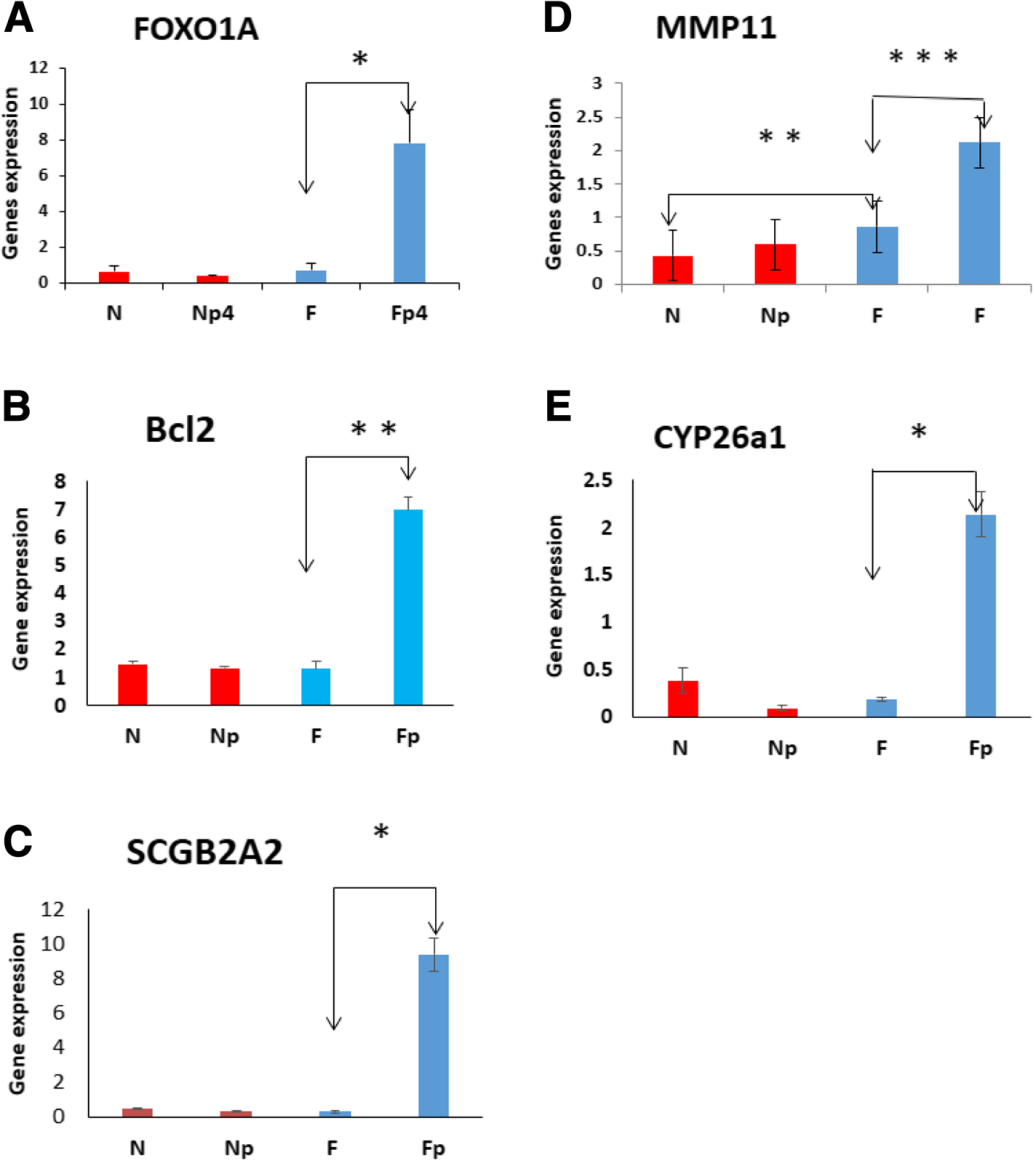
Gain of induction in response to P4. The expression levels of five genes including *FOXO1A* (**a**), *Bcl2* (**b**), *SCGB2A2* (**c**), *MMP11* (**d**), *CYP26a* (**e**) were determined by qRT-PCR after treatment with P4 (1.0 ng/ml) for 3 days in PrMyoN and PrMyoF cells. (*N* = PrMyoN cells without treatment, Np = PrMyoN cells after treatment with P4, F = PrMyoF cells without treatment, Fp = PrMyoF cells after treatment with P4). These experiments were performed with 10 different cultured cell specimens. Five genes show either decrease or no change in response to P4 in MyoN cells. However, in PrMyoF cells, all the 5 genes including *Foxo1A* (**a**), *Bcl2* (**b**), *SCGB2A2* (**c**), *MMP11* (**d**), *CYP26a1*(**e**), exhibited a significant upregulation in response to P4 treatment. * *p* < 0.05, **p < 0.01. ***p < 0.001

In MyoF primary cells, significant upregulation of five genes (*Bcl2*, *FOXO1A, SCGB2A2, CYP26a1 and MMP11*) was observed in response to P4 treatment (Fig. 2). As shown in Fig. 2a, although the *FOXO1A* gene showed no difference of RNA expression between MyoN and MyoF cells at basal levels, and no significant change was found in prMyoN cells in response to P4 treatment (*P* = 0.5), significant gain in induction was observed in prMyoF primary cells in response to P4 treatment (*P* < 0.05).

The basal levels of *Bcl 2* gene expression between MyoN and MyoF primary cells did not reach significant difference (*P* = 0.7). However a significant increase of *Bcl2* expression was observed in MyoF cells in response to P4 treatment (*P* < 0.01) (Fig. 2b), but not in MyoN cells. Similar finding was achieved for *SCGB2A2* gene as MyoF cells exhibited gain in induction of *SCGB2A2* gene expression (*P* < 0.05) but not MyoN cells in response to P4 treatment (Fig. 2c).

For MMP-11, a significant differential expression between MyoF and MyoN (*p* < 0.05) was observed. MyoN cells showed insignificant gain in induction in response to P4 treatment. However, MyoF cells exhibited a significant gain in induction after P4 treatment (*P* < 0.01) (Fig. 2d).

*For CYP26a1* gene a significant gain in induction in MyoF was observed in response to P4(*P* = 0.05) (Fig. 2e). But gain in induction of *CYP26a1* gene expression was not found in Pr MyoN cells .

### Genes show gain of repression in response to progesterone (P4)

**Fig. 3:**
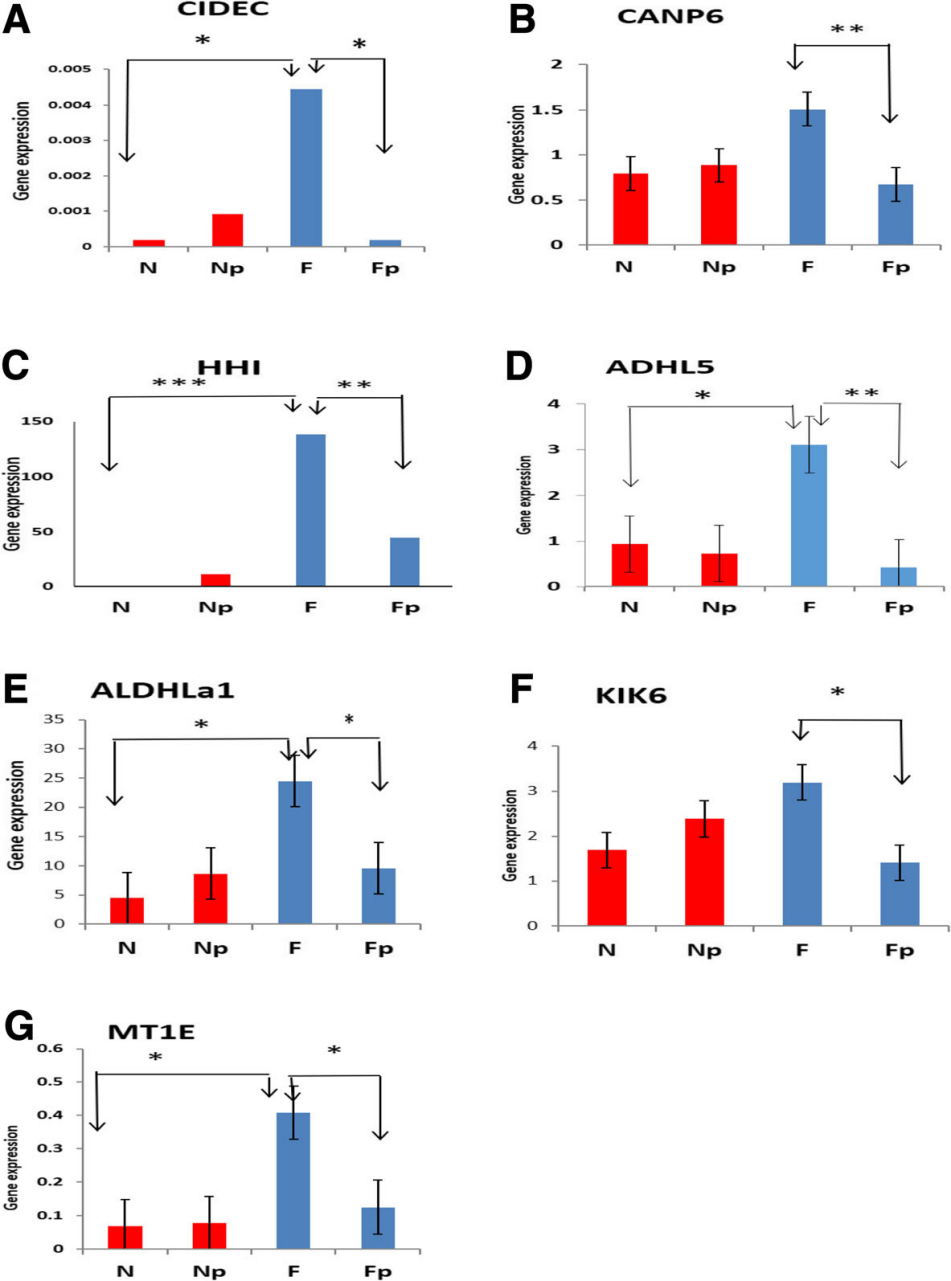
Gain of repression in response to P4. The expression of seven genes *CIDEC* (**a**), *CANP6* (**b**), *HHI* (**c**), *ADHL5* (**d**), *ALDH1a1* (**e**), *KIK6* (**f**), *MT1E* (**g**) was determined by q-RTPCR in Pr MyoN and PrMyoF cells after P4 treatment (1.0 ng/ml) for 3 days. *N* = PrMyoN cells without treatment, Np = PrMyoN cells after adding P4, F = PrMyoF cells without treatment, Fp = PrMyoF cells after adding P4. These experiments were performed with 10 different cultured cell specimens. * *p* < 0.05, ** *p* < 0.01. *** *p* < 0.001

The other seven genes showed down regulation in MyoF cells in response to P4 treatment. Three of these genes are responsible for apoptosis and cell cycle. As shown in (Fig. 3a), the expression of *CIDEC* gene was significantly higher in PrMyoF as compared to PrMyoN cells (*P* < 0.05). In addition, P4 treatment resulted in a significant gain of repression in MyoF cell (*P* < 0.05), but not in MyoN cells. Also the gain of repression in respond to P4 was also found for *CANP6* gene (Fig. 3b) (*P* < 0.05) and *HHI* gene (*P* < 0.05)(Fig. 3c) in MyoF cells, but not in MyoN cells. Comparing the basal levels of *CANP6* and *HHI* expression between PrMyoN and PrMyoF, the expression of *CANP6* gene exhibited no different but the expression of *HHI* gene exhibited statistically significant higher in PrMyoF cells as compared to PrMyoN cells (*P* < 0.001).

The RNA expression of 2 genes (*ADHL5*, *ALDH1A1*) related to RA synthesis enzymes was also measured at basal levels as well as in P4-treated MyoN and MyoF cells. For *ADHL5* gene (Fig. 3d), significant differential response to P4 was found between PrMyoN and PrMyoF cells. A significant gain in repression in PrMyoF cells after P4 treatment (*P* < 0.05), but not in PrMyoN cells. Similar finding was observed for *ALDH1a1 expression* in response to P4 in PrMyoF and PrMyoN cells (Fig. 3e).

The expression of gene *KIK6* (Fig. 3f), which is the genet hat associated with regulation of inflammatory process, exhibited no difference PrMyoN and PrMyoF cells. And alteration of its expression was not observed after treatment with P4 in PrMyoN cells. However, a significant gain in repression after P4 treatment was seen in PrMyoF cells (*P* < 0.05).

**Fig. 4:**
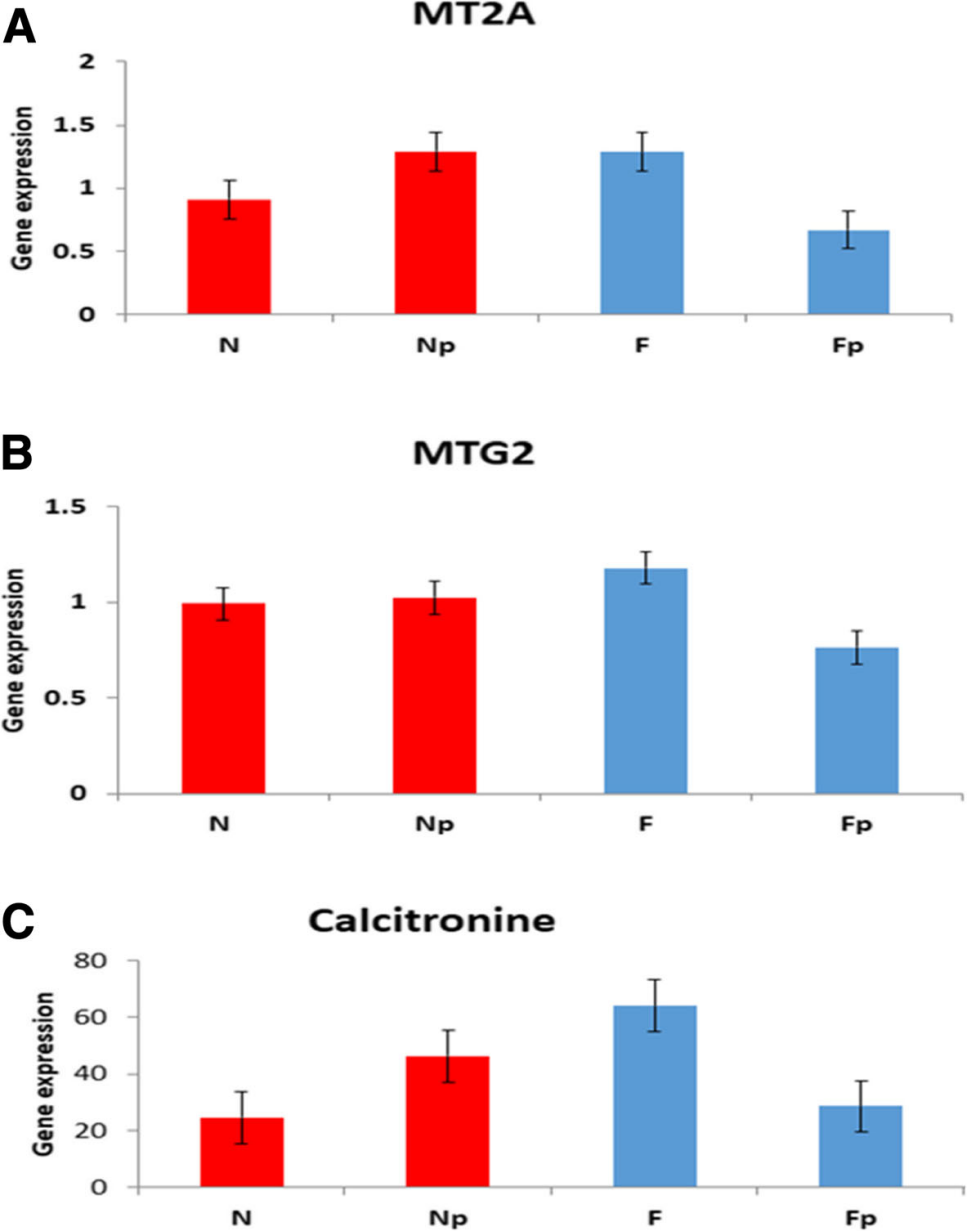
The expression of *MT2A*, *MTG2* and *Calcitonin* genes in response to P4. The expression of three genes including MT2A (**a**), MTG2 (**b**) and calcitonin (**c**) in PrMyoN and PrMyoF cells was determined by real-time PCR after P4 treatment (1.0 ng/ml) for 3 days. *N* = PrMyoN cells without treatment, Np = PrMyoN cells after adding P4, F = PrMyoF cells without treatment, Fp = PrMyoF cells after adding P4. These experiments were performed with 10 different cultured cell specimens

Metallothionein (MT) family is responsible for binding to heavy metal ions and minimize reactive oxygen species. The response of several genes of MT family to P4 were examined (Figs. 3g and 4). *MT1E* (Fig. 3g) exhibited significant repression in its expression with P4 treatment in the PrMyoF cells (*P* < 0.05), but not in PrMyoN cells. However, no significant changes of other MT family genes including MT2A and MTG2 were observed between PrMyoN and PrMyoF cells at basal levels as well as in response to P4 treatment (Fig. 4a, b).

The basal level of Calcitonin expression between PrMyoN and PrMyoF cells and their response to P4 was examined. There is no significant difference of RNA expression between MyoN verse MyoF cells. Furthermore, no significant difference of RNA expression in PrMyoN and PrMyoF cells in response to P4 treatment were found (Fig. 4c).

## Discussion

P4 is a key hormone, which contributes to the UF pathogenesis. However, the molecular mechanism by which P4 promotes the UF development is largely unknown. In this study, we used PrMyoN and PrMyoN cell model system and characterized the expression pattern of P4 response genes in response to P4 treatment, which may contribute to increased risk of UF development.

Previous study showed that cultured UF cells had an increased response to P4 compared to cultured normal myometrial cells [30]. This study also showed that P4 receptor mRNA is highly expressed in UF cells as compared the cells from adjacent myometrium. In our study, we focused on P4 response in primary cells from normal myometrium (MyoN) and at-risk myometrium (MyoF). We demonstrated that the expression of PR was higher in PrMyoF as compared to PrMyoN cells. The differential response of PrMyoN and PrMyoF to P4 seems to be attractive. Among the genes we detected, we found two types of changes in response to P4 treatment, gain in induction and gain of repression respectively. These results suggested that the network of P4/PR signaling was varied between PrMyoN and PrMyoF and the primed PrMyoF turned out to be hyper-sensitive to P4, which might lead to increased risk of UF development.

In this study, the expression of 15 P4-responsive genes was examined in PrMyoF and PrMyoN cells using q-PCR analyses. Five of these genes including *FOXO1A, CYP26a1, SCGB2A2, MMP11 and Bcl 2* showed significant up regulation in response to P4 treatment. The other seven genes exhibited a significant down regulation, these genes include *CANP6, MT1E, ADHL5, Aldh1a1, KIK6, HHI, CIDEc*. However, the expression of *MT2A*, *MTG2* and *calcitrone* was not altered in response to P4 treatment.

### Expression of genes control the apoptosis in response to progesterone

Apoptosis is a morphologic pattern of cell death [31]. There are multiple genes responsible for regulating this process. Korsmeye [32], reported that the *Bcl-2* proto-oncogene has the ability to block apoptotic cell death in multiple contexts. Increase in expression of *Bcl-2* in transgenic models will result in evasion of normal cell death mechanisms leading to accumulation of cells and tumor formation [33].

Previous studies showed that *Bcl-2* protein expression was predominant in UF cells compared to that in normal myometrium cells [34]. The expression of *Bcl-2* protein in normal myometrium cells was very low that raised the possibility that normal myometrium cells may be more susceptible to apoptotic cell death. In addition, UF cells exhibited increased expression of *Bcl-2* protein in response to P4 treatment. But the expression of *Bcl-2* protein in cultured normal myometrium cells was not affected by P4 treatment. Here in our study *Bcl-2* gene expression in at-risk myometrium tissues from the uterus with UFs was remarkably augmented by P4 treatment and this change was not found in normal myometrial cells.

Another gene that responsible also for apoptosis is *FOXO1A*, it is a member of the *FOXO* subfamily of Forkhead transcription factors [35]. According to the previous study, activated *FOXO* proteins induced expression of genes that encode for proteins involved in cell cycle inhibition [36]. Our study showed that this gene exhibited hyper-response in PrMyoF cells after P4 treatment. Another study determined the progestin effect in *FOXO1* expression and its activity in the endometrium during endometrium menstrual cycle. They showed that progestin enhanced *FOXO1* mRNA levels in mid- and late-secretory endometrium [37]. In addition, *FOXO1A* was considered as a key transcription factor responsible for mediating apoptosis of decasualized human endometrial stromal cells (HESC) in response to progesterone withdrawal during the menstrual cycle by inducing the cell death. Moreover, this study explains the effect of admission of medroxyprogesterone acetate (MPA, a synthetic progestin) in enhancing the expression of *FOXO1A* in differentiating human endometrial cells. MP also simultaneously induced cytoplasmic retention and inactivation of this gene. Withdrawal of the MPA from decidualized HESCs resulted in rapid nuclear accumulation of *FOXO1A* gene, therefore leading to activation of apoptosis and cell death [37]. Similar finding was observed in PrMyoF cells, where the expression of *FOXO1* was markedly increased in response to progesterone treatment, which provide a favorable condition for the pathogenesis of UFs.

*SCGB2A2* (Secretoglobin family 2A member 2) was considered as uteroglobin-related protein, which controls cell cycle and DNA replication. It was originally detected by differential RNA expression levels in Breast Cancer biopsies [38]. Previous studies demonstrated the effect of *SCGB* overexpression on cell proliferation in other human diseases and ovarian carcinoma. The role of *SCGB2A2* in patho-physiology of the ovarian tumor was identified [39]. The overexpression of *SCGB2A2* is positively correlated with the FIGO stage, the tumor grade and the mitotic index of the ovarian cancer [40]. In our study, although no significant expression of *SCGB2A2* was observed in ProMyoF and PrMyoN cells, ProMyoF cells was remarkably augmented by P4, which was not the same with ProMyoN cells. This study suggests that P4 might promote the UF development by increased cell proliferation and enhancement of the DNA replication via *SCGB2A2*.

The cell death-inducing DFF45-like effector (CIDE) family includes *CIDEa, CIDEb*, and *CIDEc*. It has been reported that the (CIDE) family plays an important role in lipid and fat metabolism [40–42]. Previous studies have reported that *CIDEa, CIDEc* were highly expressed in adipose tissue, and in skeletal muscle. I*CIDE*c is capable of inducing apoptosis in mammalian cells [43]. DFF45 is a subunit of the DNA fragmentation factor which is cleaved by active caspase-3 during apoptosis. The main function of *CIDEC* is energy homeostasis, and its absence may result in insulin resistance, and resistance to diet-induced obesity [44]. Here in our study this gene showed gain of repression in response to P4 as a marker of decrease in the apoptosis that might be involved in UF development.

Another gene that showed gain in regression in our study was *CANP6.* It is calcium-activated cysteine proteinases. Calpains have been involved in many biological events including regulation of the cell cycle, apoptosis, cell adhesion and motility [45, 46]. So the regression of this gene will decrease the apoptosis as well as down regulation of cell cycle all together will favor the development of UFs.

### Expression of genes control the retinoic acid in response to progesterone

RA, is the natural metabolite of vitamin A. Previous studies showed that RA signaling played an important role in the female reproductive trace function [47]. *ADH5* and *ALDH1a1* are RA synthesis enzymes and *CYP26a1*(cytochrome P450, family 26, subfamily a, polypeptide1) is a RA catabolizing enzyme. Previous studies demonstrated that the expression of these are altered during preganyc which may be related to progesterone signaling*. ADH5* expression was increased by 2.5 folds during pregnancy. The expression of *ALDH1a1* in the endometrial glandular compartment was increased on gestational early days until the implantation phase. The expression of *CYP26a1 was* strongly detected in the uterine epithelium. Moreover, these studies indicated that early pregnancy needed the synthesis and degradation of RA to be balanced to allow RA signaling to prepare for implantation without harmful effects on the embryo [48]. Our result has demonstrated that RA synthesis genes (*ADH5, ALDH1a1*) show gain in repression in response to P4, and RA catabolic enzyme (*CYP26a1)* were rapidly gain induction by the P4-PR axis. This might result in increasing retinoic acid catabolism and decrease in Vitamin A in the myometrium tissue. All this together will favorable the proliferation of the myometrium, which provide pro-fibroid condition to increase the risk of UF development.

### Expression of other genes in response to progesterone

In human, over 20 functional Matrix metalloproteinases *MMPs have been* identified [48]. *MMPs* are zinc endopeptidases capable of releasing the growth factors that are bound to the extracellular matrix (ECM) [49] regulating cell-matrix and cell-cell interactions. Matrix metalloproteinase 11(*MMP11)* is responsible of serpins cleavage and so it stimulate the development of *tumor* [50, 51]. Our study showed that *MMP-11* mRNA was significantly increased in myometrium cells from uterus with UFs compared with myometrium cells of normal one. Also *g*ene expression of *MMP11* showed significant gain in induction after P4 treatment in PrMyoF cells. Previous study demonstrated increased expression of *MMP-11* in UFs as compared to myometrium.

*KLK6* belonging to kallikreins gene family is a serine protease [52–54]. It is down-regulated by the P4-PR axis. *KLk6* is responsible for regulation of the inflammatory process and vascular permeability, and edema [55]. Previous studies in mouse graved uterus showed that this gene was upregulated by the P4-PR axis signaling suggesting the important role of this gene in the implantation of the embryo in the uterus [27]. In our study, the repression of this gene in response to P4 may result in the formation of the UFs by losing the regulation of the inflammatory process.

Indian hedgehog *(HHI*), one of the Hedgehog family of ligands, is a P4-regulated gene in the uterus [56, 57]. It plays a role in down regulation of cellular division [58]. In the human endometrium, the role of Hedgehog signaling in UFs is largely unknown [59]. *HHI* gene shows a significant decrease in expression s between the early secretory to the mid secretory phase. It also plays a role in embryo implantation by regulation of stromal cell proliferation and inhibition of epithelial E2 signaling. In addition, Hedgehog signaling was involved in the women with endometriosis [60–62]. and in endometrial cancer [63]. Here in our study this gene showed a gain of repression in the PrMyoF cells in response to P4, suggesting that this change may be involved in the increased risk of UF pathogenesis.

Metallothioneins *(MTs*) comprise a family of genes clustered on chromosome 16q that bind to heavy metal ions and minimize reactive oxygen species. Previous studies demonstrated low MT expression in endometrium of women with endometriosis [29]. In our study *MT1E i*s the only one we detected that showed significant repression with P4 treatment in the PrMyoF cells.

The location of the Calcitonin gene is in non-neuronal tissues. Its define function remains unclear, but previous studies identified their role in cardiovascular system as it exhibited a potent vasodilator effect [64]. There are many other researches done on calcitonin effect on the heart. These researchers show its role in prevention of ischemia as well as endotoxic shock [65]. These shock can be done by the suppressor effect of calcitonin on some pro-inflammatory cytokines production [e.g., macrophage inflammatory protein-2 (*MIP-2*) and keratinocyte chemoattractant (KC)] [66, 67]. Moreover, calcitonin has a protective effect against ischemia [68]. So the decrease in its expression may result in ischemia stimulation of the inflammatory reaction. However, in our study it showed gain in repression suggesting the complex role of this gene in response to P4 treatment.

## Conclusion

Our studies demonstrate for the first time that PrMyo cells and PrMyoN from either at risk myometrium or normal myometrium exhibit a differential response to P4, the key hormone for UF development. P4-responsive genes in PrMyoF cells exhibit a P4-hyper-responsiveness, suggesting that myometrium from uterus with UFs is primed and become at risk for later tumorigenic transformation. However, due to sample size (*n* = 10) and race limitation (all from Caucasian), further investigating the role of P4 in alteration of normal myometrium in a large sample size as well as using tissues from at high risk populations such as African American are needed. Moreover, **e**valuating of P4 response in relevant animal model or 3D system is also highly needed for understanding the pathogenesis of UFs.

## Data Availability

The datasets used and/or analyzed during the current study are available from the corresponding author on reasonable request.

## Abbreviations

DFF: DNA fragmentation factor
ECM: Extracellular matrix
FBS: Fetal bovine serum
GnRH agonists: Gonadotropin releasing hormone agonist
HESC: Human endometrial stromal cells
KC: Keratinocyte chemoattractant
LNG IUS: Levo –norgestrine intra-uterine device
MMSC: Myometrial stem cell
MPA: Medroxyprogesterone acetate
MyoF: Human myometrial cells from uteri with fibroid
MyoN: Human myometrial cells from normal uteri
P4: Progesterone
PR: Progesterone receptor
PRGs: Progesterone-regulated genes
PrMyoF: Primary human myometrial cells from uterus with fibroids
PrMyoN: Primary human myometrial cells from normal uterus
qPCR: Quantitative real-time pole maries chain reaction
RA: Retinoic acid
SmBM: Smooth muscle cell basal medium
SPRM: Selective progesterone receptor modulator
UFs: Uterine Fibroids
WB: Western blot
